# Haemolytic activity of soil from areas of varying podoconiosis endemicity in Ethiopia

**DOI:** 10.1371/journal.pone.0177219

**Published:** 2017-05-11

**Authors:** Jennifer S. Le Blond, Peter J. Baxter, Dhimiter Bello, Jennifer Raftis, Yordanos B. Molla, Javier Cuadros, Gail Davey

**Affiliations:** 1 Department of Earth Sciences, Imperial College London, London, United Kingdom; 2 Core Research Labs, Natural History Museum, London, United Kingdom; 3 Institute of Public Health, University of Cambridge, Cambridge, United Kingdom; 4 Department of Work Environment, University of Massachusetts Lowell, MA, United States of America; 5 The Queens Medical Research Institute, University of Edinburgh, Little France, Edinburgh, United Kingdom; 6 Department of Earth Sciences, Addis Ababa University, Addis Ababa, Ethiopia; 7 Wellcome Trust Centre for Global Health Research, Brighton and Sussex Medical School, University of Sussex, Brighton, East Sussex United Kingdom; 8 Department of Earth Sciences, Natural History Museum, London, United Kingdom; US Geological Survey, UNITED STATES

## Abstract

**Background:**

Podoconiosis, non-filarial elephantiasis, is a non-infectious disease found in tropical regions such as Ethiopia, localized in highland areas with volcanic soils cultivated by barefoot subsistence farmers. It is thought that soil particles can pass through the soles of the feet and taken up by the lymphatic system, leading to the characteristic chronic oedema of the lower legs that becomes disfiguring and disabling over time.

**Methods:**

The close association of the disease with volcanic soils led us to investigate the characteristics of soil samples in an endemic area in Ethiopia to identify the potential causal constituents. We used the in vitro haemolysis assay and compared haemolytic activity (HA) with soil samples collected in a non-endemic region of the same area in Ethiopia. We included soil samples that had been previously characterized, in addition we present other data describing the characteristics of the soil and include pure phase mineral standards as comparisons.

**Results:**

The bulk chemical composition of the soils were statistically significantly different between the podoconiosis-endemic and non-endemic areas, with the exception of CaO and Cr. Likewise, the soil mineralogy was statistically significant for iron oxide, feldspars, mica and chlorite. Smectite and kaolinite clays were widely present and elicited a strong HA, as did quartz, in comparison to other mineral phases tested, although no strong difference was found in HA between soils from the two areas. The relationship was further investigated with principle component analysis (PCA), which showed that a combination of an increase in Y, Zr and Al_2_O_3_, and a concurrent increase Fe_2_O_3_, TiO_2_, MnO and Ba in the soils increased HA.

**Conclusion:**

The mineralogy and chemistry of the soils influenced the HA, although the interplay between the components is complex. Further research should consider the variable biopersistance, hygroscopicity and hardness of the minerals and further characterize the nano-scale particles.

## Introduction

Podoconiosis (from the Greek podos meaning foot, and konion meaning dust) is a form of non-infectious elephantiasis found in tropical regions, such as the African Highlands and Central America (e.g., [1],[2]), commonly in those involved in arable farming. Podoconiosis is thought to occur after prolonged barefoot exposure to irritant soils formed from volcanic deposits ([3],[4],[5]) and it is considered a major health problem, contributing a substantial economic burden and causing widespread social stigmatisation in endemic countries.

Evidence suggests that podoconiosis is due to an abnormal inflammatory reaction to soil particles entering the lower legs of genetically susceptible individuals ([6]). The mechanism by which soil particles pass through the skin of the sole of the foot and the pathological changes that lead to the disease are unknown, but the view of Price [4] who undertook the first comprehensive studies, mainly in Ethiopian populations, was that quartz in the soil gained entry to the lymphatics and provoked inflammation with subsequent fibrosis that caused obstruction of lymphatic flow in the legs. This view was widely accepted after Prices’ death, even though it remains as a hypothesis. In its early stages the disease has been found to be readily treatable by wearing shoes and improving foot hygiene, which can halt progression and even reverse the chronic oedema, but without intervention the oedema becomes persistent and often progressive with characteristic and deforming skin changes in the lower legs and feet. The attribution of the disease to quartz particles in the cultivated soil does not explain its specific geographical distribution, because quartz is widely present in most soils and Price himself was the first to document that podoconiosis in Eastern Africa was mainly confined to highland areas and then almost exclusively to where the soil was of volcanic origin.

In addition to quartz, several papers hypothesize that the disease is linked to the presence of clay minerals ([7]), organic matter or elevated levels of Zr and Be ([8]) in the soils. Price ([4]) found inorganic ‘microparticles’ (described as being 0.5–2 μm in diameter, and mostly silicates) in the lymphatic tissues in both patients with and without podoconiosis, a statistically significant difference was observed in the form of the phyllosilicate within the lymph tissues of podoconiosis patients by analysing the elemental ratio of Al/Si from the particles. To further this work, Molla et al. ([9]) used multivariate statistical analyses (combining data from a disease prevalence survey and the characterization of surface soils, both from a region in northern Ethiopia) to identify the components within the soil that strongly associates with the presence of podoconiosis. In this analysis, strong correlations between the quantities of the phyllosilicate mineral smectite and the disease were observed.

**Fig 1:**
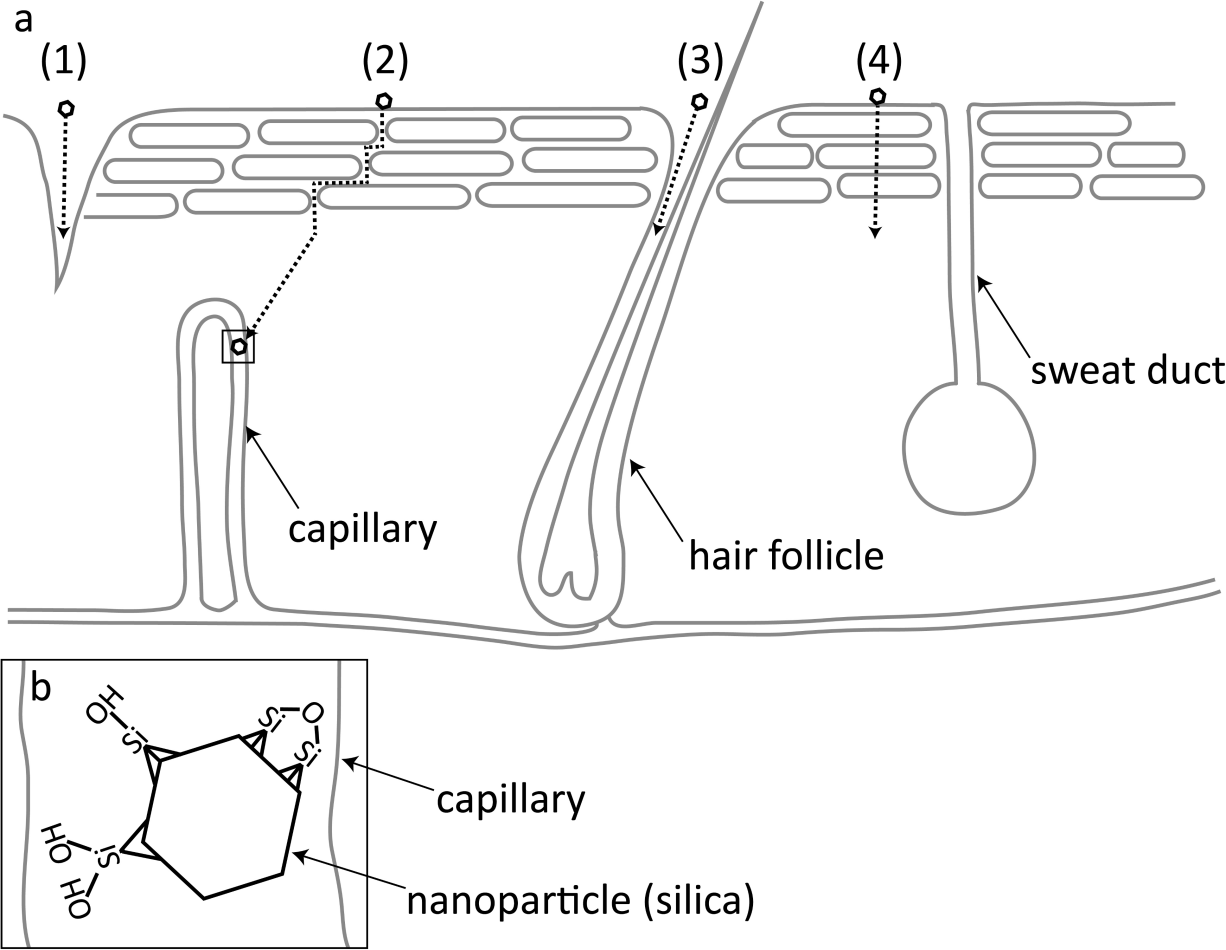
Potential nanoparticle penetration routes through the skin. A) 1) via broken epidermis, 2) intercellular route, 3) transappendageal route via hair follicles (or sweat ducts) and 4) intracellular route (modified from [10]) B) magnified schematic of a silica nanoparticle in the capillary, showing example surface features that are thought to contribute to HA in vitro.

The progression of podoconiosis is likely to be due to constituents within the soil entering into the human body, either via skin penetration or movement through compromised skin (Fig 1). Intact, healthy skin acts as an efficient barrier to protect the human body from foreign substances. However, it is thought that nanoparticles ≤4 nm in diameter, which equates to the maximum intercellular space, can penetrate and permeate the skin barrier (e.g., [10]). In addition to the intercellular route, nanoparticles between 2 and 20 nm may be able to penetrate the stratum corneum, reaching viable epidermis, via sweat glands and hair follicles ([11]). This appears to be the most efficient route of penetration and permeation of large organic molecules and nanoparticles. Experimental data shows that nanoparticles >20 nm can pass through disrupted or compromised skin ([12], [13],[14],[15],[16]). When considering podoconiosis, previous authors have hypothesised that solutions containing dissolved silicates (originating from the alkaline tropical soils) and particulates penetrate the skin then enter the cells where the pH is much lower ([17]). The dissolved solutions may be released into the components of the cell, where a concentration threshold is reached and re-precipitation occurs. However, the stratum corneum in podoconiosis patients is often compromised and visibly damaged and it is likely that cracked, compromised skin allows sufficiently small soil particles to enter into the dermal layer ([18]). Furthermore, it has been shown that people with podoconiosis have significantly lower levels of hydration in their outer layer of skin (stratum corneum), which is likely to lead to cracking and splitting ([19]). Prolonged and repeated exposure to the abrasive components of the volcanic soil (e.g., quartz) could exacerbate stratum corneum degradation and enable nano- and submicron particles to penetrate the viable epidermis.

The in vitro HA of micro-silica particles has been traditionally regarded as in some way predictive of the in vivo activity of inhaled quartz particles in the lung and the development of silicosis and lung cancer in workers exposed to silica dusts (e.g., [20]). Haemolysis has previously been used to test the potential cytotoxicity of mineral particles, such as silica (e.g., [21]; [22]) and asbestos (e.g., [23]) and correlations between the haemolytic effect and pathogenicity have previously been reported in animal experiments ([24]). Biological membranes are important targets of silica toxicity and the haemolysis assay that uses red blood cells (RBC) can be used as a ‘first look’ at toxicity as it is relatively inexpensive to undertake. Furthermore, Pavan et al. ([25]) noted a statistically significant correlation between the HA of silica particles and pro-inflammatory cytokine release (interleukin 1ß), which implies that similar physico-chemical properties involved in RBC rupture may also be implicated in the processes that lead to pro-inflammatory responses. Interest has now widened to consider the predictive power of the haemolysis assay to commercial nanoparticles, including materials with biomedical applications containing nanostructured amorphous silica (e.g., [25]).

The use of the haemolysis assay to predict the potential pathogenicity of respirable particles of volcanic ash containing crystalline silica (a mineral known to induce a high haemolytic response when tested as a single-phase mineral) has, however, highlighted some important issues. For example, a positive haemolytic response was observed when crystalline silica-containing volcanic ash samples from Mount St Helens, USA and Soufriere Hills volcano, Montserrat, were tested (e.g., [26],[27]), but the haemolysis assay protocol utilised sheep erythrocytes that are known to be more fragile than those from humans (e.g., [28]). Subsequent assessments of the haemolysis assay (e.g., [29]) have determined that based on the quantity of crystalline silica in the volcanic ash the expected haemolytic response is likely moderated and masked by other mineral phases and/or components. In addition, in their detailed study Pavan et al. ([30]) noted that the HA varied between silica polymorphs (varying in properties, such as crystalline and amorphous) and speculated that the mechanism behind HA involved the surface properties of the silicas, namely the distribution of silanols and siloxane groups. Hence, the interaction between highly haemolytic components such as crystalline silica and other minerals/phases within a heterogeneous mixture, for example components within volcanic ash or soil, can modify the bio-reactivity and result in a HA that is unexpectedly reduced. With this in mind, further research is needed to explore the potential bio-reactivity of natural mixed-phase samples, such as well-characterised soils, to establish the components that may be contributing to the HA.

Although a role for quartz in the pathogenesis of podoconiosis cannot be excluded, our investigations have been focused on other constituents of volcanic soils that could be responsible for initiating or promoting the disease process. Smectite and kaolinite clays are commonly present in soils derived from the weathering of eruption products, and these behave in vitro, like quartz, in eliciting a strong haemolytic activity. Clays also constitute the finest fraction of soils and are even present as nanoparticles. In this study we explored the role of the haemolysis assay in the initial assessment of the bio-reactivity of soils in areas of the Ethiopian Volcanic Plateau endemic and non-endemic for podoconiosis to determine if this assay has a place in the study of podoconiosis etiology or in designating land areas at elevated risk. We used soil samples that have been previously characterized in terms of their mineralogy and chemical composition ([9],[31]). In addition, we present the results from grain size analysis. The combined data set was analysed to determine whether there was a correlation between the haemolytic response of the soil and podoconiosis prevalence (from [32]). Furthermore, we explored the correlation between the haemolytic potential and the characteristics of the soil (specifically mineralogy, geochemistry and particle size).

## Methods

### Sample collection and preparation

Surface soil samples (n = 103) were collected from within the Gojjam region of the Ethiopian Volcanic Plateau (EVP), from an area approximately 30 km^2^. At each site, approximately 1 kg of the surface soil (to a depth of ~5 cm) was collected using a plastic trowel and placed into sterile, airtight sample bags, then double bagged and labelled. The sampling was carried out on private land, and we confirm that the owner of the land gave permission to conduct the study on this site (permission was given orally). The GPS waypoint and elevation were recorded for each sample site. The study area encompasses two zones of podoconiosis endemicity: Yewla, an area in which the disease can affect up to 18% of the local village population, and Rob Gebeya where less than 1% of the local village population is affected by the disease. The prevalence study is detailed in Molla et al. ([32]), but briefly, data on health and socioeconomic status was gathered from a house-to-house survey of 611 individuals that had been selected by a case-control protocol. Samples of soil were taken from a randomised selection of cases and controls, and of the 103 soils collected initially, we categorised 81 samples by disease endemicity status—40 from areas regarded as ‘endemic’ and 41 from areas regarded as ‘non-endemic’ ([9]) to enable comparison of soil characteristics and haemolytic potential. Of the 103 soil samples, 22 were from areas of unknown endemicity, and were included in the study to increase the sample size in the multivariate analyses on the data without considering endemicity.

Once in the laboratories at Addis Ababa University, the soil was air dried, homogenised and aliquots were separated. Samples were then shipped to the Natural History Museum (NHM), UK, for analysis (under the soil licence no. 7172/198480/1, held at the NHM). Once in the UK, further sub-samples of the soil were separated using a riffle sample divider. Aliquots of the soil were dried at 105°C for at least 24 hours, lightly hand ground with a mortar and pestle, and sieved to <2 mm.

### Soil sample characterisation

As part of a concurrent study, the soil samples were characterised according to their bulk chemical composition (major oxides) and mineralogy ([31]). The main mineralogical phases identified (using semi-quantitative X-ray diffraction) were quartz, feldspars, poorly crystalline Fe oxides, smectite- and kaolinite-like clays, mica, chlorite and amorphous silica. In this study, the minor elements within the soil samples were determined by lithium metaborate digestion and inductively coupled plasma–atomic emission spectroscopy (ICP-AES) analysis at the NHM (using the methodology detailed in [9]). In addition, the total carbon content in the soil samples was determined with a Thermo Finnigan EA112 elemental analyzer, also based at the NHM.

### Reference soil samples

As the composition of the soil samples was determined by XRD, the following pure-phase reference minerals (PPRMs) were sourced, from collections at the NHM, to represent dominant phases within the samples: calcite, goethite, hematite, quartz, microcline and the following phyllosilicates: kaolinite, interstratified kaolinite-smectite, smectite and chlorite. The PPRMs were characterized in the same fashion as the samples.

### Particle size and morphology

A Malvern Mastersizer laser diffractometer with a Hydro Mu attachment, based at the University of Cambridge was used for the hydrodynamic size analysis of the particles that make up the soils and standard mineral samples (the latter selected according to the results of the XRD characterisation). Before analysis, the soils were dried at room temperature, after which they were gently crushed and dry sieved (using 2 and 1 mm mesh size sieves). The organic material in the soil was removed by adding 5 mL H_2_O_2_ (30% w/w) and heating to 65°C for 1 hour. The soil samples were analysed twice, first using water as a dispersant and then after pre-treatment to encourage de-flocculation of the clay minerals (e.g., [33]). The de-flocculation process involved immersing the soil samples in sodium hexapyrophosphate (4.4 w/v %) for >3 hours (gently agitating) in a water bath at 90°C. The suspension was then centrifuged at 3500 rpm for 15 min and the supernatant discarded leaving the sediment at the base of the tube. Before the sample was added to the Malvern Mastersizer for analysis, water was added to the samples and the suspension was agitated with a glass rod on a vortex mini shaker. For the measurements, the refractive index of the material was set to 1.53 and an absorption coefficient of 0.1 was used ([34]). Water was used as a dispersant, with a pump speed of 2250 rpm, ultrasonic time 60 sec, obscuration was set at 15–20% and a measurement time of 20 sec. Averages of three readings were taken for each sample, given as volume percent that was then converted into cumulative volume percent.

The morphology of the particles within one soil sample was investigated using a JEOL 2000FX transmission electron microscopy (TEM) with EDS analysis at Imperial College London. The soil samples were suspended in deionised water, shaken and left to settle for 2 minutes before an aliquot was taken and dropped onto a copper grid. The TEM was operated at 100 kV and 10 nA beam current.

### Zeta potential

Surface charge, the Zeta potential (ζ), was determined on a NanoMalvern (Malvern Instruments). The soil samples were prepared as at 1 mg mL^-1^ suspensions in deionised water (pH 7.4), and subjected to ultrasonication for 12 min prior to analysis. The temperature of the analysis was kept at 21°C, and triplicate analyses (each with 12 runs) were averaged for each sample.

### Specific surface area

Specific surface area analysis was carried out by Brunauer, Emmett and Teller (BET) nitrogen adsorption on a Micromeritics Gemini analyzer at the Natural History Museum, London, UK. Before analysis, all samples were degassed under a continuous N_2_ flow at 100°C for at least 12 hours (e.g., [35]). Each sample was analyzed at least three times and the result was averaged.

### Haemolysis assay

The soil samples and standard mineral samples were subjected to the haemolysis assay as a method of assessing one pathway of toxicity in vitro. Erythrocytes were isolated from whole citrated blood taken from a healthy volunteer, with written informed consent under the full institutional ethical approval of the University of Edinburgh (where the haemolysis assay took place). The blood was prepared according to previously described methods in Lu et al. ([36], but briefly the RBCs were isolated and added to a buffer (NaHCO_3_ (30 mM), NaHPO_4_ (16 mM), dextrose (110 mM), mannitol (55 mM), diluted in deionized H_2_O). For each assay, the buffered RBC were washed three times with saline and centrifuged at 4000 rpm for 5 min. Thereafter, aliquots of 200 μL RBC were added to 3.8 mL saline to make the final concentration of 5% by volume RBC in saline for use in the assays.

The soil samples were weighed, suspended in saline and sonicated for 10 min. 150 μL of the particle suspension (in triplicate) was added into each well of a 96-well flat bottom plate, and each sample was analysed in three separate experiments. Negative controls consisting of 150 μL of saline and positive controls consisting of 150 μL of 0.1% Triton-X 100 were also added to each of the sample plates. 75 μL of the particle suspension was added to each of the wells and gently mixed by pipetting. The plates were incubated at room temperature for 30–40 min, shaking gently. Each soil was tested over a range of concentrations, 0.0625 to 1 mg mL^-1^. Then the plates were centrifuged at 2500 rpm for 5 min, after which 75 μL of the supernatant was removed from each well and transferred to a clean 96-well plate. The absorbance of the plate at a wavelength of 550 nm was read in a spectrophotometer and the percentage haemolysis was calculated using the following equation:

$$\%\;haemolysis\;(x)=\frac{\left[optical\;density-negative\;control\;optical\;density\right]}{\left[(positive\;control\;optical\;density-negative\;control\;optical\;density)*100\right]}$$ (1)

Where the negative control optical density is the optical density measured from the saline, and the positive control optical density is that measured from the Triton-X 100 solution. Both the positive and negative controls were included, as a triplicate, in each one of the sample plates. Hence the % haemolysis could be adjusted for each plate reading, and each session, to account for potential instrument drift. In addition, the soil was also analysed as a suspension in the buffer solution, without the addition of RBCs to test for possible interferences due to light scattering and the particles’ own absorption properties. The haemolysis assays were carried out over a number of days, in three different sessions (June 2010, March 2012 and December 2012). The haemolytic response measured from the internal standards (i.e. samples of known haemolytic response) DQ12 quartz and the TiO_2_ (rutile) was found to vary marginally with time. In order to control for variations in the haemolytic response, caused for example by lamp degradation over time in the spectrophotometer, the haemolytic response was normalized to the positive control (DQ12 quartz) measured on the same day that the assay was carried out.

For each soil material, a concentration (mg mL^-1^) vs. response (% cell lysis) curve was generated. Because these curves were largely sigmoidal in shape, the steepest slope in the linear region of the curve (where the slope of % cell lysis per unit soil concentration is the greatest), was recorded and used in all subsequent analyses. This is the recommended methodology for comparing response data across multiple materials ([37],[38]). Furthermore, the slopes were normalized to that of quartz DQ12, a well understood haemolytic material.

Data analysis was performed using non-parametric statistical tests, Student’s t-test, Spearman’s Rho correlation coefficient, multivariate linear regression and principal component analysis (PCA) in IBM® SPSS statistics program (versions 20 and 21). A value of p <0.05 was considered significant.

## Results

### Characteristics of the soil

The soils collected from the Ethiopian Volcanic Plateau were fully characterized, in terms of their mineralogy and chemistry, as part of a concurrent study (see supplementary information). The summary statistics for the samples are shown in Table 1, where data are presented as the geometric mean, geometric standard deviation (GSD = 84^th^ %-ile/50^th^ %-ile of the cumulative number distribution) and range, for all of the soil samples, and divided into the endemic and non-endemic categories (indicating the presence or absence of podoconiosis, respectively). For comparison, the mean (arithmetic) soil compositions for major oxides and elements from soils sampled in Sub-Saharan Africa, are also shown in Table 1 (from [39]). The majority of the major oxides and elements found within the soil in this study are similar to the reported median or within the range of values documented for soils in Sub-Saharan Africa. The exception is aluminium oxide, which is higher than the reported range (at 19.5 wt. %, geometric mean), where the reported range for Al_2_O_3_ in Sub-Saharan Africa is up to 16.8 wt. %. The content of silicon dioxide is lower in the samples of soil analyzed here than that from Sub-Saharan Africa. The low SiO_2_ content in our soils maybe due to a combination of several factors, including underlying geology (predominantly mafic), and altitude (where there is little detrital input, wind-derived or riverine, which would increase the quartz content of the soil).

**Table 1:** Summary of the chemistry and mineralogy of the soils, presented for all soil samples together and by endemicity.

		All soil samples (*n* = 103)				Average soil composition from Sub-Saharan Africa^a^		Soils from endemic areas (*n* = 40)			Soils from non-endemic areas (*n* = 41)		
		Geometric Mean	Median	GSD	Range	Median	Range	Geometric Mean	GSD	Range	Geometric Mean	GSD	Range
**Geographical**	**Altitude (m)**	2528.5	2471.0	1.2	1949–3592	-	-	2222.0	1.0	2147–2943	2851.4	1.1	2183–3592
**Chemical composition (oxides in wt. %, elements in ppm)**	**Al**_**2**_**O**_**3**_	19.5	19.9	1.1	8.6–25.1	6.5^a^	0.02–16.8^a^	20.3	1.1	18.4–25.1	19.3	1.1	15.2–23.7
	**CaO**	0.5	0.5	1.5	0.2–2.7	0.3^a^	0.01–59.7^a^	0.5*	1.3	0.2–1.3	0.6*	1.5	0.3–2.7
	**Fe**_**2**_**O**_**3**_	14.4	13.9	1.3	4.6–23.0	2.8^a^	0.03–23.4^a^	13.6	1.1	11.5–18.1	16.0	1.3	11.-21.8
	**K**_**2**_**O**	1.3	1.4	1.2	4.6–23.0	1.0^a^	0.04–9.4^a^	1.4	1.1	1.0–1.8	1.1	1.3	0.4–2.9
	**MgO**	0.9	0.9	1.3	0.4–1.7	-	-	0.8	1.1	0.6–1.2	1.1	1.2	0.7–1.7
	**MnO**	0.3	0.3	1.2	0.1–0.6	0.04^a^	0.0002–0.8^a^	0.3	1.1	0.2–0.4	0.4	1.2	0.3–0.5
	**Na**_**2**_**O**	0.2	0.2	1.6	0.1–1.7	-	-	0.2	1.1	0.1–0.3	0.2	1.5	0.1–1.7
	**P**_**2**_**O**_**5**_	0.4	0.5	1.5	0.1–1.5	0.01^a^	0.006–0.5^a^	0.4	1.3	0.2–0.8	0.6	1.9	0.3–1.5
	**SiO**_**2**_	42.8	43.3	1.1	30.9–73.4	-	-	45.1	1.1	35.6–50.3	39.3	1.1	30.9–48.3
	**TiO**_**2**_	2.5	2.5	1.3	0.3–4.1	0.5^a^	0.0004–4.3^a^	2.5	1.1	2.2–3.0	2.8	1.3	1.6–4.1
	**Ba**	340	323	1.4	93–855	-	-	296	1.1	204–509	399	1.4	279–718
	**Cr**	53	165	1.2	1–695	45^a^	0.7–598 ^a^	188*	1.1	147–365	113*	1.4	1–695
	**Ni**	40	109	1.3	1–181	12 ^a^	0.3–364 ^a^	133	1.1	99–174	82	1.5	1–181
	**Sc**	8	26	1.2	1–48	-	-	13	1.1	1–31	14	1.3	1–48
	**Sr**	11	43	1.5	1–84	47 ^a^	1.2–1985 ^a^	20	1.2	1–85	21	1.4	1–84
	**Y**	51	53	1.2	29–190	9.2 ^a^	0.2–109^a^	58	1.1	45–74	41	1.3	29–60
	**Zr**	400	386	1.2	247–1459	-	-	427	1.1	346–518	349	1.1	259–448
**Elemental concentration (%)**	**total carbon**	2.6	3.0	1.6	0.2–10.2	-	-	2.8	1.4	0.2–5.7	3.9	1.6	0.7–10.2
**Mineralogical composition (%)**	**iron oxide**	22.0	21.5	1.2	6.2–39.9	-	-	21.1	1.1	17.3–28.8	24.0	1.3	17.7–39.9
	**quartz**	10.8	12.5	1.9	0.1–44.9	-	-	14.2*	1.6	3.0–29.1	8.5*	1.7	0.1–44.9
	**Amorphous silica**	14.5	14.7	1.2	9.3–38.5	-	-	14.2*	1.2	9.3–25.7	15.5*	1.2	10.7–24.2
	**feldspars**	0.1	0.05	1.0	0.1–41.3	-	-	0.06	1.0	0.1–10.7	0.1	290.8	0.1–30.2
	**kaolinite**	15.3	15.8	1.9	1.8–50.1	-	-	15.7*	2.0	6.3–36.0	12.9*	2.1	2.5–35.4
	**smectite**	0.9	0.7	3.6	0.1–30.6	-	-	1.0*	2.6	0.3–5.6	0.8*	2.6	0.1–5.5
	**mica**	17.1	21.2	1.7	0.1–46.4	-	-	26.4	1.3	9.7–44.6	16.4	1.4	0.1–40.0
	**chlorite**	0.4	0.05	88.0	0.1–11.0	-	-	0.09	13.5	0.1–2.3	1.5	2.4	0.1–10.2

Data from

^a^[39]

*Not significantly different (α = 0.05)

The data between the endemic and non-endemic regions were compared, and the results are shown in Table 1. The elevation at which soil samples were collected was statistically significantly different for endemic areas (averaging ~2220 m above sea level (asl)) and non-endemic areas (~2850 m asl). There were statistically significant differences in the bulk chemical composition of endemic and non-endemic soils, including major oxides, minor elements and total carbon, with the exception of CaO and Cr. Likewise, the mineralogy of endemic and non-endemic soil samples was statistically significantly different for iron oxide, feldspars, mica and chlorite, but not for quartz, amorphous silica, kaolinite and smectite.

**Fig 2:**
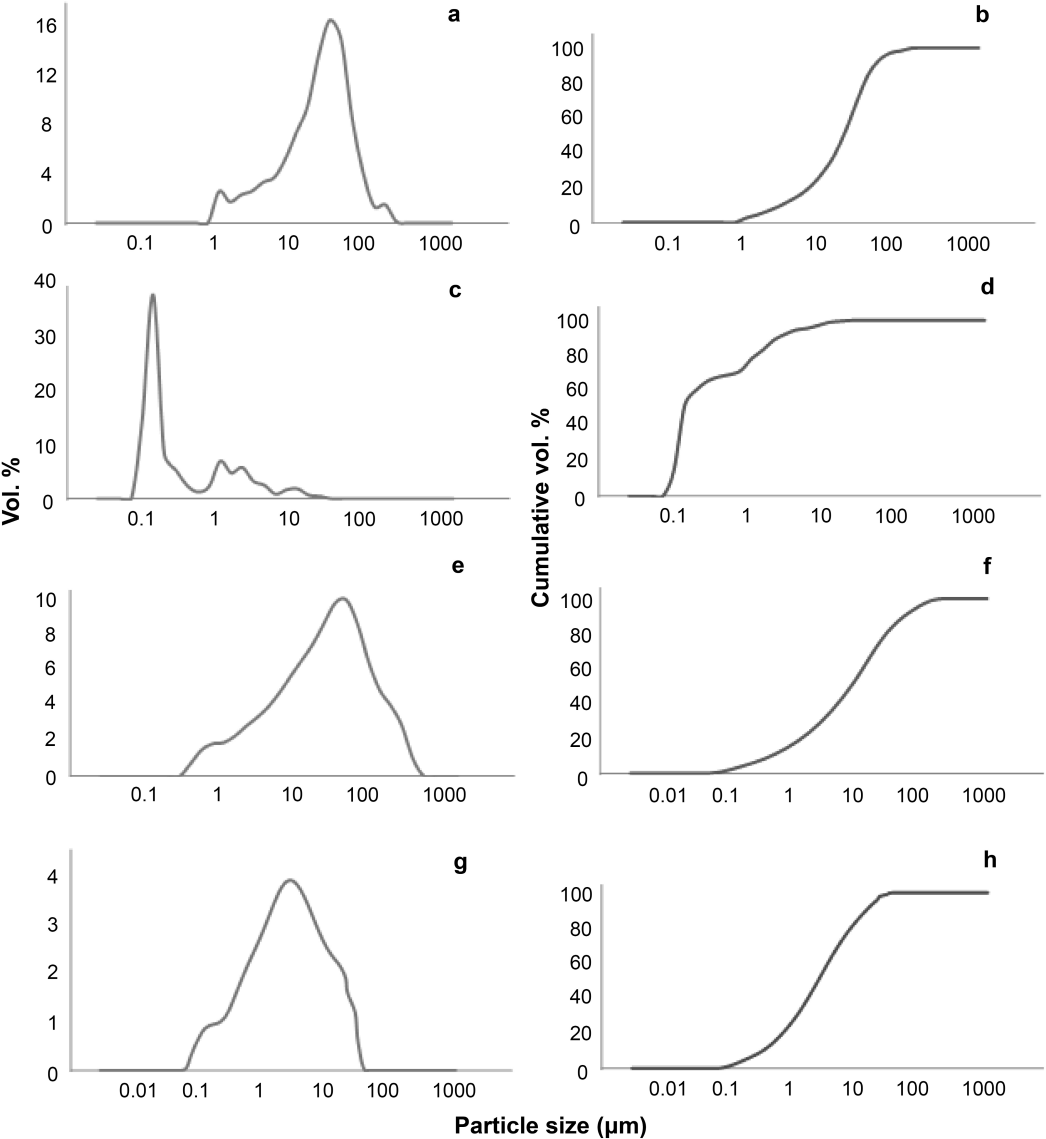
Size distribution curves for sample DM-SE SO 22B and SO 4003. (A) and (E) were analysed in water (C) and (G) with deflocculant. Figs (B), (D), (F) and (H) represent the cumulative curved of (A), (C), (E) and (G), respectively.

Particle size analyses, using two methods of preparation, are given in Table 2. As the size distribution was typically right-skewed (i.e. non-normal), the distributions were characterized by geometric mean, geometric standard deviation and range. The volume % of particles was calculated for the size fractions <500 nm, <1 μm and <10 μm. The measured particle size was highly dependent on the sample preparation method (water or deflocculant). For example, the proportion of <500 nm (aerodynamic) diameter particles in the soils ranged from 0.03% when dispersed in water, and 0.34% when pre-treated in the deflocculant, an order of magnitude increase. Fig 2 shows the frequency distribution curves (absolute and cumulative) of the particle size determined for two soil samples (DM-SE SO 22B and SO 4003), chosen to best represent the two extremes of the particle size data. The size distribution curve for DM-SE SO 22B (Fig 2A and 2B) dispersed in water shows a predominant peak and a corresponding smooth cumulative distribution curve. The curve for DM-SE SO 22B pre-treated with deflocculant (Fig 2C and 2D), however, shows a multi-modal distribution, in which a large proportion of the particles were <1 μm in diameter, and a second minor group is within 1–10 μm. Sample SO 4003 shows a different particle size distribution. The size distributions for the water-dispersed sample (Fig 2E and 2F) and that treated with deflocculant (Fig 2G and 2H) were similar in shape but the distribution is displaced to lower particle size in the latter. When considering the soils from endemic and non-endemic areas, the particle size measured in the soils pre-treated with deflocculant was statistically significantly different (Table 2): the soils from endemic regions had a greater proportion of particles in each of the size categories (<500 nm, <1 μm and <10 μm; i.e., lower particle size than soils from non-endemic areas). However, the particle size distributions measured in water were not statistically significantly different between soils from endemic and non-endemic areas.

**Table 2:** Summary of the particle size data for the soil samples, presented for all soil samples together and by endemicity.

Particle size		All soil samples (*n* = 103)			Soils from endemic areas (*n* = 40)			Soils from non-endemic areas (*n* = 41)		
		Geometric mean (vol. %)	GSD (vol. %)	Range (vol. %)	Geometric mean (vol. %)	GSD (vol. %)	Range (vol. %)	Geometric mean (vol. %)	GSD (vol. %)	Range (vol. %)
**Water**	**<500 nm**	0.03	1.5	0.0–2.0	0.95*	1.3	0.0–2.0	0.09*	1.3	0.0–1.5
	**<1 μm**	1.70	1.4	0.1–8.0	2.35*	1.3	0.2–8.0	2.35*	1.3	0.1–7.3
	**<10 μm**	27.52	1.2	16.9–41.6	26.55*	1.2	18.9–34.6	28.40*	1.3	16.9–41.6
**Deflocculant**	**<500 nm**	0.34	8.3	0.0–68.0	1.71	18.3	0.0–68.0	0.13	4.4	0.0–8.4
	**<1 μm**	7.29	3.3	0.4–72.1	11.50	3.6	2.8–72.1	4.45	2.5	0.4–37.4
	**<10 μm**	59.78	1.5	14.6–98.6	60.84	1.6	37.5–97.7	53.72	1.3	32.2–97.9

*Not significantly different (α = 0.05).

**Fig 3:**
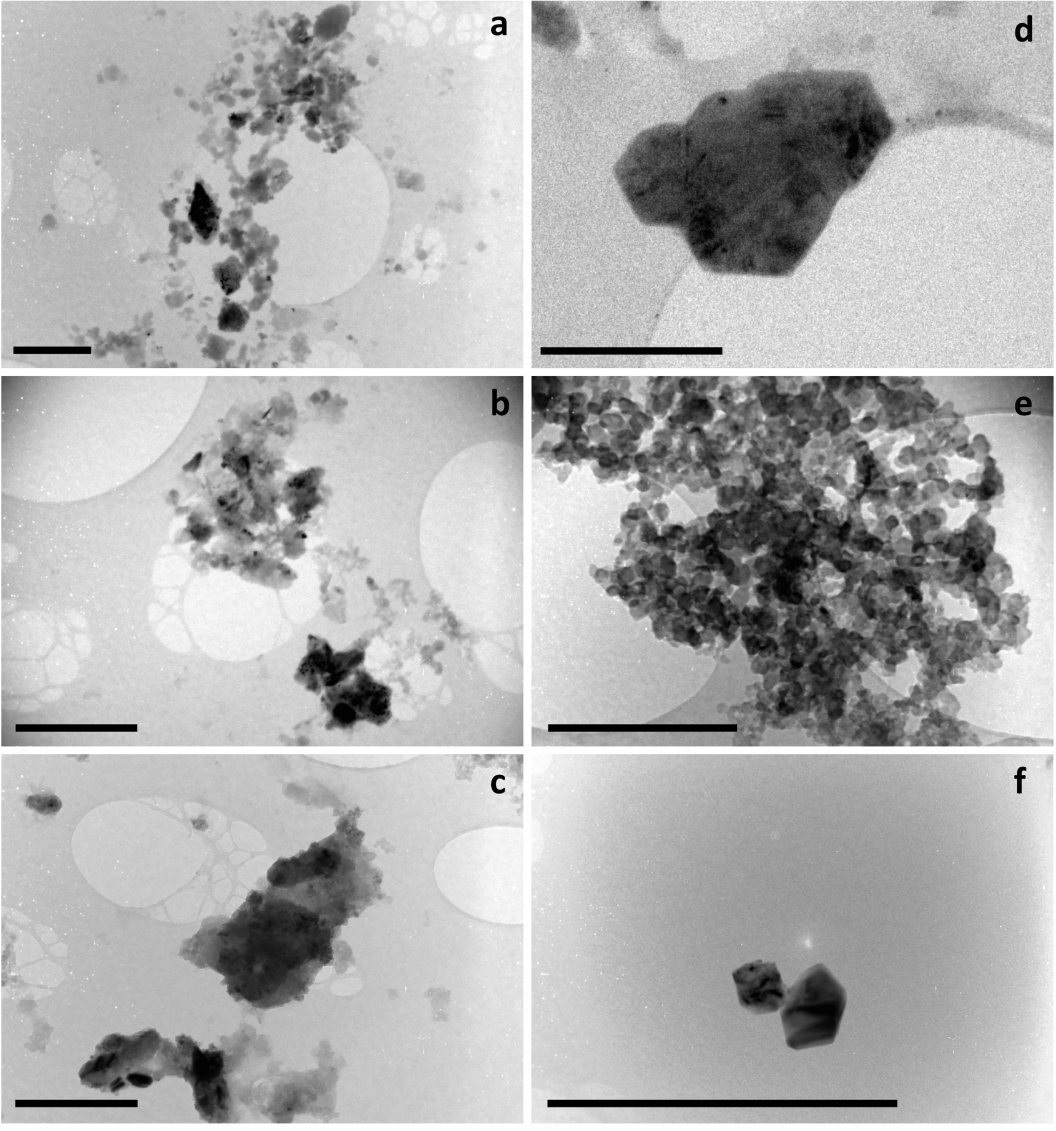
Transmission electron microscope (TEM) images from soil sample SO 6117 from an endemic region. (A), (B) and (C) show aggregations of sub-micron particles, and soil sample SO 4111 from a non-endemic region (D), (E) and (F) showing well-formed polygons and agglomerated spheres. The polygons and agglomerations of spheres are likely kaolinite and iron oxide, respectivley (EDS analysis, not shown). Scale bars are 0.5 μm.

The particle morphology was investigated by TEM in two samples of soil, one from the endemic and one from the non-endemic regions (SO 6117 and 4111, respectively; Fig 3). The particles displayed a variety of shapes, from rounded to polygon shapes. The particles were seen to agglomerate and cluster together and many particles within the nano-size range (<100 nm) were observed (Fig 3B). Clusters of particles were both monodisperse (Fig 3B) and polydisperse (i.e. a measure of the heterogeneity of the particle size distribution). EDS analysis (n = 35) indicated that the majority of the particles analyzed were either phyllosilicates (characteristically kaolinite-rich, Fig 3D and 3F) or iron oxide (Fig 3E) in composition.

The zeta potential (ζ) of a random selection of soil samples (n = 15) had a geometric mean of– 28.3 mV and ranged from -24.23 to -32.4 mV (data not shown).

We investigated possible correlations between particle size and mineralogical composition in the samples. We found similar relationships between the particle size and mineralogy for the soil samples suspended in water and deflocculant, except for mica and amorphous silica. There was a positive relationship between the quantity of mica and amorphous silica, and the proportion of particles measured as <1 μm in water (r = 0.533 and 0.455, respectively), however the relationships for both these minerals was negligible for mica and negative for the amorphous silica when a deflocculant was used to disperse the sample. This suggests that the mica and amorphous silica control the particle size when the soils are dispersed in water, but they exert little influence on the particle size when the samples are pre-treated with a deflocculant. The relationship between kaolinite, and the proportion of particles <1 μm in water is negative (r = -0.348) and positive in the deflocculant (r = 0.226). This suggests that the amount of kaolinite influences the proportion of particles <1 μm in in the deflocculant to a greater extent than in water, as the deflocculant acts to disaggregate the particles. The same relationship, however, is not observed for smectite.

The total carbon content of the soil is negatively correlated with the proportion of particles <1 μm in deflocculant (r = -0.424), indicating that the carbon within the soil is not preferentially within the sub-micron fraction. The amount of Ni, Sc and Sr are all positively correlated (r = 0.529, 0.794 and 0.736, respectively) with the proportion of particles <1 μm in water but this relationship is negligible for particles <1 μm in deflocculant. This indicates that the mineral phases containing these elements are predominantly found in the mineral phases that do not disaggregate on treatment with the deflocculant. This is confirmed by the fact that both Sc and Sr positively correlate with amorphous silica (r = 0.564 and 0.409, respectively) and mica (r = 0.467 and 0.591, respectively), none of which phase is expected to disperse with deflocculation treatment. In addition, Ni correlates positively with mica (r = 0.580). There is a weak positive correlation between the amount of Zr and the proportion of particles <1 μm in deflocculant (r = 0.395), which becomes negligible with <1 μm in water. Some of the Zr is likely, therefore, to be contained within the fraction of soil that disaggregates in the deflocculant. Zr is positively correlated with SiO_2_ (r = 0.635), and is not correlated positively with any other mineral phases, indicating that zircons are likely present.

### Characteristics of the standards

The characteristics of these PPRMs are detailed in Table 3. The quantity of particles below 1 μm from the deflocculant method was consistently greater than or equal to the quantity of particles from the water-dispersion method. The quantity of particles below 1 μm, for both the water and deflocculant method, were higher for the phyllosilicate minerals (kaolinite, smectite, kaolinite-smectite). The ζ measurements for all PPRMs, prepared at 1 mg mL^-1^ in deionised water, were predominantly negative (Table 3) and varied between -6.8 and -63.7 mV (calcite and quartz, respectively). The exception was hematite that was positive (1.7 mV). Typically, ζ for the phyllosilicates was more negative than for the non-phyllosilicates.

**Table 3:** Summary of the characteristics of the pure-phase reference minerals used in this study, including specific surface area (SSA), particle size (in water and deflocculant), Zeta Potential (ζ) and the haemolytic potential (given as the slope of absorbance versus soil suspension concentration).

Sample	Chemical formula	Notes	SSA (m^2^ g^-1^)	<1 μm in water (vol. %)	% <1 μm in deflocculant (vol. %)	ζ (mV)	Haemolytic activity (HA) (slope,)	HA normalised to DQ12 control
**calcite**	CaCO_3_	C Cuff	2.5	2.3	2.5	-6.8	6.1	1.0
**chlorite**	(Mg,Fe,Al)_6_(Al,Si)_4_O_10_(OH)_8_	CCa-2 Ripidolite, El Dorado County, Ca, USA	2.7	0.01	0.1	-28.0	1.0	0.2
**goethite**	FeO(OH)	BM1985,650	11.8	2.5	2.3	-6.9	0.4	0.1
**hematite**	Fe_2_O_3_	BM58073	7.3	0.6	0.6	1.7	4.3	0.7
**kaolinite**	Al_2_Si_2_O_5_(OH)_4_	API 17 Montana (Pure Geochem Lab IC)	21.4	2.9	14.6	-55.1	8.0	1.3
**kaolinite-smectite**	Al_2_Si_2_O_5_(OH)_4_-(Na,Ca)_0,3_(Al,Mg,Fe)_2_Si_4_O_10_(OH)_2_•n(H_2_O)	CWP 73 S, from S Hillier	61.6	4.1	50.4	-50.4	275.7	45.2
**microcline**	KAlSi_3_O_8_	Low temp, specimen 8487	1.4	0.8	0.7	NA	1.0	0.2
**quartz**	SiO_2_	Pre-powdered Min-U-Sil	1.2	0.6	0.7	-63.7	2.5	0.4
**smectite**	(Na,Ca)_0,3_(Al,Mg,Fe)_2_Si_4_O_10_(OH)_2_•n(H_2_O)	Na-montmorillonite, Bella Fourche	72.5	0.9	45.6	-30.6	657.4	107.8

The specific surface area for the PPRMs ranged from 72.5 to 1.2 m^2^g^-1^ (smectite and quartz, respectively). There is a good correlation (R^2^ = 0.98) between the specific surface area and proportion of particles measured below 1 μm in the deflocculant.

### Haemolytic activity

**Fig 4:**
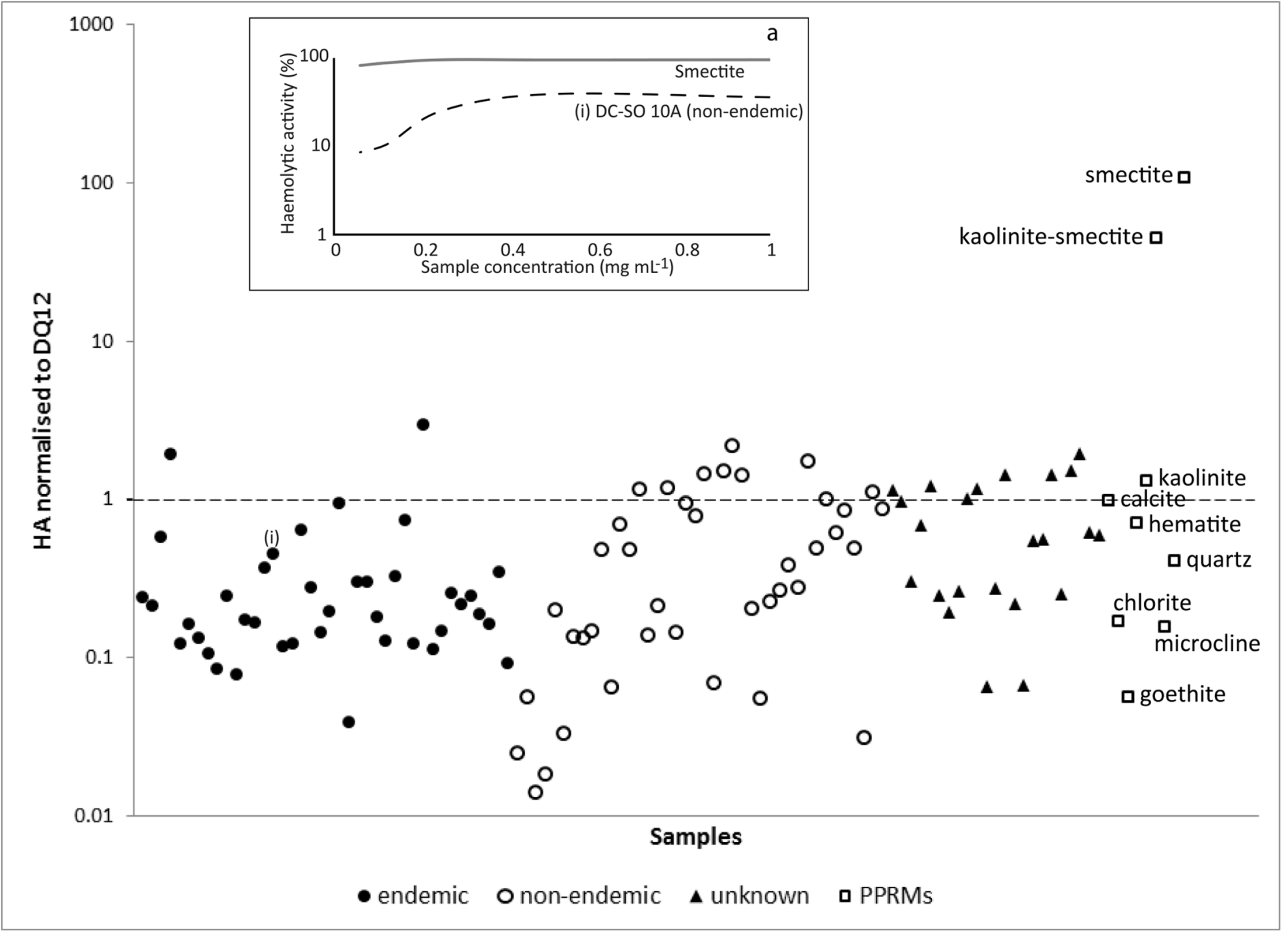
HA of the soil samples (shown in terms of their endemicity) and PPRMs, presented as the slope of the HA normalised to DQ12. 9A) shows the haemolysis curve for two selected samples.

The HA presented as the steepest slope of the linear range of the HA vs. mineral concentration curve) of the soil samples was lower than that of DQ12 quartz. After normalization of the soils HA to that of DQ12 quartz, the geometric mean of the HA for all the soils was 0.28. The maximum haemolytic value was 2.9 and the minimum was 0.01. Eighteen soils had a normalized HA value greater than that for DQ12 quartz, the positive control (Fig 4). Of these 18, 2 were from endemic, 8 from non-endemic, and 7 from areas of unknown endemicity.

**Fig 5:**
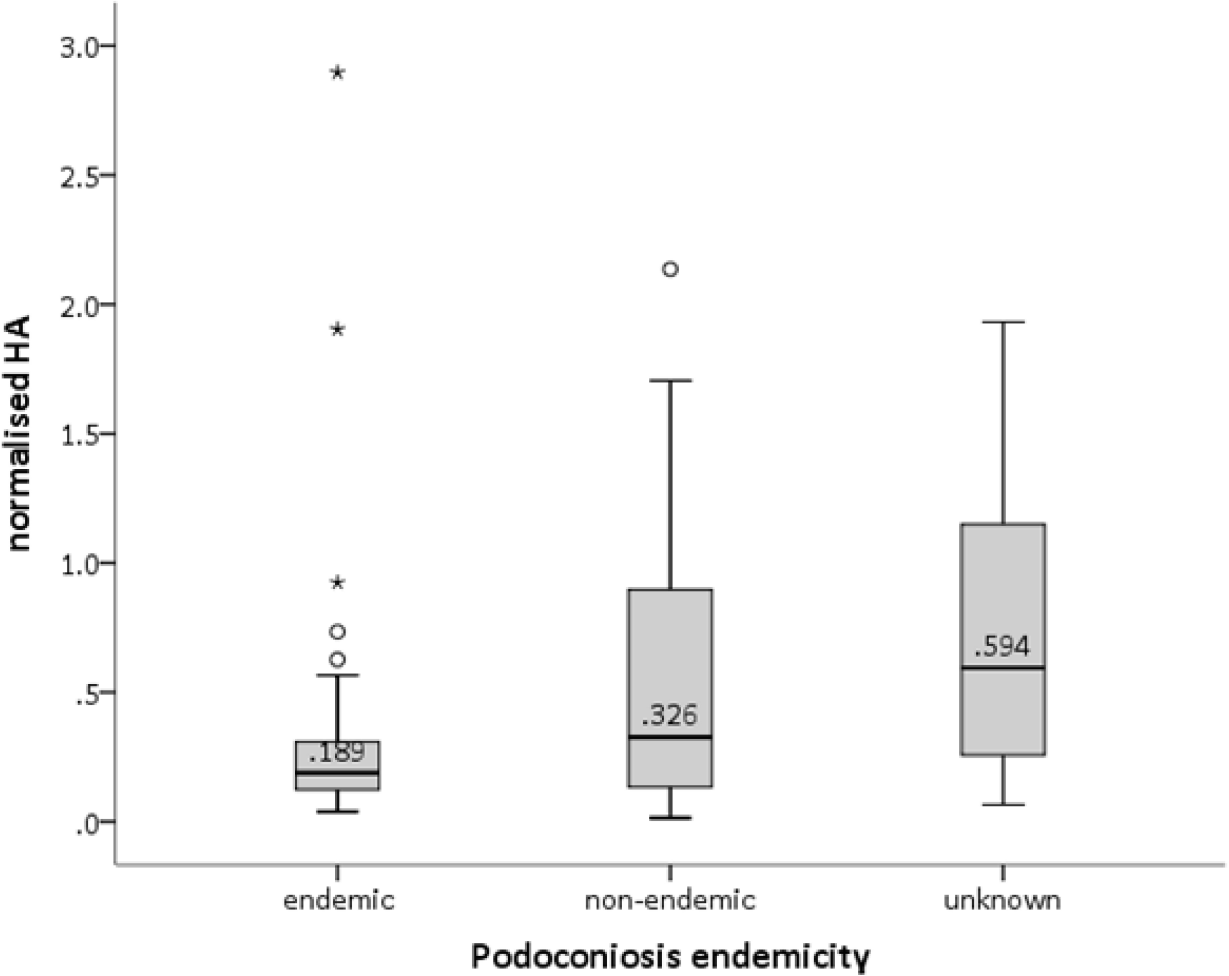
Box and whisker plot for normalised HA for the soil samples from podoconiosis-endemic, -non-endemic and areas with unknown endemicity.

The median normalised HA from the endemic soils was lower than that for the non-endemic soils (0.189 and 0.326, respectively, Fig 5). However, the normalised HA response elicited from the soils was not statistically significantly different between the three areas of endemic, non-endemic, and unknown endemicity.

The HA for the PPRMs was as follows: smectite > kaolinite-smectite > kaolinite > calcite > hematite > quartz > chlorite > microcline > goethite (Table 3). The normalized haemolytic potential for the PPRMs to DQ12 quartz (normalized HA; DQ12 is a positive control), produces high values for smectite and kaolinite-smectite (107.8% and 45.2%, respectively; Fig 4). Interestingly, the quartz sample (Min-U-Sil) used as a PPRM in this study does not elicit as high a haemolytic response as the DQ12 quartz. For the PPRMs, there was a strong correlation between the specific surface area and the normalized HA (r = 0.92), and between the proportion of particles below 1 μm in diameter (measured using the deflocculant) and the normalised HA (r = 0.86).

When the soil samples were analysed, without the addition of RBCs, the average readings from the spectrometer were not above that of the blanks analysed (data not shown). There was, however, limited correlation between the normalised HA and the iron oxide determined by mineralogy and that determined by chemical analysis (r = 0.34 and r = 0.37, respectively).

As the mineralogy of the soil samples and the HA activity of the individual mineral phases (PPRMs) are known, a prediction of the HA for the soils can be made by multiplying the known HA of the PPRMs by the proportion of each mineral within each sample. The experimental normalised HA was then compared to the calculated HA, and the Spearman’s Rho correlation was low (r = 0.262, sig. = 0.008). Thus, mineralogy alone cannot explain the HA results. A more complex analysis was carried out in which the calculated HA (from mineralogy) and the other variables, such as proportion of particles <1 μm (water and deflocculant) and total carbon, were incorporated. The multivariate regression analysis was performed to predict normalized HA generated the following model, with an R^2^ = 0.324, F(2,100) = 24.01, sig. = 0.0005 (Eq 2).

$${HA}_{normalized}=0.313*{HA}_{calculated}-0.450*C_{total}$$ (2)

In Eq 2, the calculated HA is positively correlated with the (experimental) normalised HA, but negatively correlated with the total carbon (organic and inorganic) in the samples.

### Association of soil characteristics and haemolysis with podoconiosis prevalence

Spearman’s rho correlation showed a significant association (r = >0.4, p = <0.01) between the normalised HA haemolytic response and the following soil characteristics; Al_2_O_3_ (r = 0.44), Fe_2_O_3_ (r = 0.37), K_2_O (r = 0.36), Na_2_O (r = -0.40), quartz (r = -0.41) and total C (r = 0.55). Kaolinite and smectite had a small positive correlation with normalised HA (r = 0.24 and 0.28, respectively). Although the phyllosilicate components of the soil are separated into single phases during the quantification (see [31]), in the studied soils kaolinite and smectite typically occur as interstratified phases in which there are layers of one and the other mineral within the crystals (interstratified clay minerals are thoroughly described in [40]). Hence, when kaolinite and smectite were added, the correlation with normalised HA was stronger than with either kaolinite or smectite phases alone; r = 0.35 (p < 0.001).

Multivariate analysis was used to assess the relationship between normalised HA and particle characteristics. The characteristics were divided into chemistry, that uses the chemical composition of the samples with the other variables (major oxides, minor elements, total carbon and particle size for both <1 μm from water and deflocculant) and mineralogy, which uses the mineralogy of the samples rather than their major chemical composition (minerals, minor elements, total carbon and particle size for both <1 μm from water and deflocculant). The variables were standardized before analysis to produce z scores (calculated as the variable value minus the mean, divided by the standard deviation, which results in a set of z scores for each variable with a mean of 0 and a standard deviation of 1) to enable direct comparison between the variable datasets. Using normalised HA as a dependent variable, a multivariate regression (forward and stepwise) was run to independently predict normalised HA from the chemistry and mineralogy characteristics. The collinearity of the variables in the regression analysis was significant, and so principal component analysis (PCA) was carried out to re-group the collinear variables into a single variable.

Several iterations of PCA were run, and all were judged on their selection criteria (e.g., conforming to the thresholds for Kaiser-Meyer-Olkin measure of sampling efficiency and Bartlett’s test of sphericity). The PCA scores were then entered into a stepwise regression model, using the normalised HA (standardised) as the dependent variable. The most successful model was for the chemical characteristics (Eq 3), with an R^2^ = 0.319, F(2,100) = 23.39, and p = 0.0005, using the regression model:

$${HA}_{normalized}=-1.235-0.564*(PC3)+0.312*(PC4)$$ (3)

**Fig 6:**
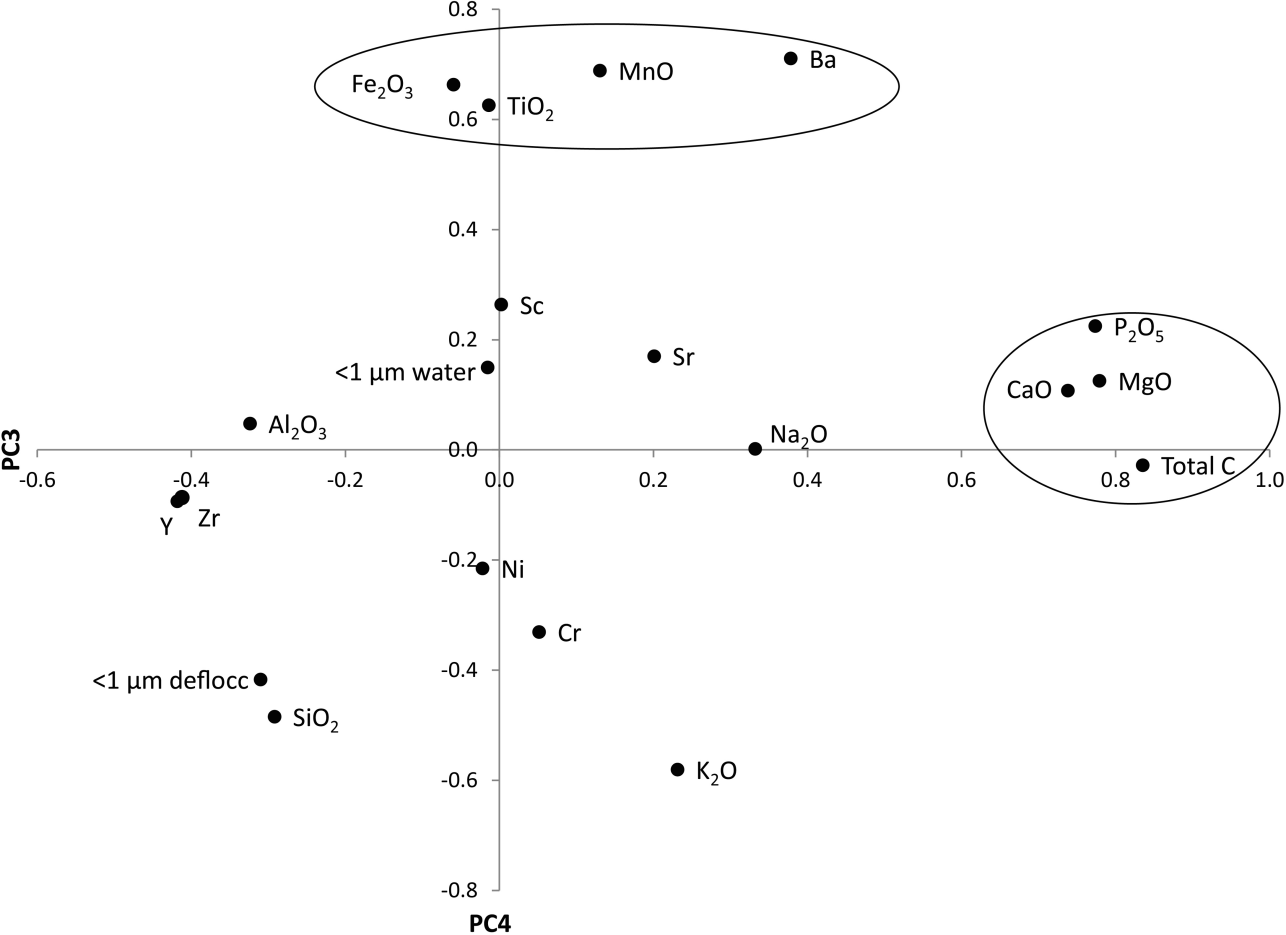
PCA loadings for the chemical characteristics (including the major oxides, minor elements, total carbon and particles <1 μm in water and deflocculant) used in the multivariate regression analysis. The circled variables are those with a strong factor score (>0.6).

The PCA scores are given in Fig 6, and the variables with component loadings >0.6 have been grouped on the two axis. The grouping on the positive PC3 axis is CaO, MgO, P_2_O_5_ and total C, and according to the regression model, the collective influence of these variables on the normalised HA is negative. Conversely, the negative loadings on the PC3 axis are Y, Zr and Al_2_O_3_, which have a positive influence on the normalised HA. The positive loadings on the PC4 axis comprise Ba, Fe_2_O_3_, MnO, TiO_2_, and these variables collectively have a positive influence on the normalised HA.

## Discussion

### Soil characteristics

In this study, the particle size distributions for the de-flocculated samples were statistically significantly different from those dispersed by water: the de-flocculated samples included a greater proportion of fine particles, whereas fine particles tended to aggregate in water. The important issue here is which of the two analyses is more representative of the dispersions produced within bodily fluids. The question is complex because 1) salt content in water first acts as a mineral dispersant and then as a flocculent as salt concentration increases, and 2) different salts or substances in solution or dispersion modify mineral dispersion in different ways ([41]). It is important to acknowledge the limitations of the laser diffraction technique for size distribution characterization of nanoparticle dispersions, in particular with regard to the influence of larger particles on light scattering and its inability to measure particle number concentration (e.g., [37]) Clay minerals tend to aggregate and stack together via electrostatic interactions and Van de Waals forces, so measuring the particle size in water may underestimate the actual particle size of the sample, especially in clay-rich samples where the particle size is reduced. The ionic strength of biological fluids, such as lymph fluid where the reactions that contribute to podoconiosis may occur, is much greater (~150 mM; e.g., [42]) than water, and hence the reactivity of clay minerals will not be the same. Considering this, additional characterization techniques (such as tunable resistive pulse sensing [43], or X-ray diffraction [44]) may provide better insights into the behaviour and stability of soil nano/particles in suspension.

The size, size distribution and dispersion stability (i.e., the re-agglomeration rate) of nanoparticles in suspension are impacted by the dispersant conditions and type of dispersant used (e.g., [37]). The dispersion protocol used here (sonication and dispersant medium) may not be optimal for our samples, as full dispersion in the finer particles may have not been achieved. However, the protocols employed were intended to mimic the behaviour of soils in the environment. To further characterize the soil components, and to gain insight into the dispersal dynamics of the particles once in the human body, a dispersant that mimics the physiological milieu should be used. In the case of podoconiosis, human blood serum or lymphatic fluid would be an appropriate medium to adopt, and the finer fraction of the soil should be isolated for analysis (and is planned future work).

During the haemolysis assay, sonication of the solution (before the RBCs are added) reduces the likelihood of particle aggregation, but it is well known that fine particles and nanoparticles agglomerate in saline solutions ([45]). The aggregation of finer particles may partially explain the presence of a bimodal distribution in the samples (e.g., Fig 2), although bimodal distributions are common in nanoparticle dispersions as the peak at larger particle size may indicate poorly dispersed particles. The particles within the soil sample were observed to be agglomerated together in the TEM, in both polydisperse and monodisperse arrangements. This may be due to sample preparation (water-dispersion and drying), although when combined with particle size data, we presume that particles will aggregate in the suspension media in the case of podoconiosis, and aggregation is more intense in the absence of stabilizers such as serum proteins at 10% w/v.

### Soil haemolytic potential

Although the HA of the soil did not correlate directly with podoconiosis endemicity, there was a moderate correlation between the proportion of smectite and kaolinite (phyllosilicates) in the soil and haemolytic potential (r = 0.275 and 0.237, respectively). Endemicity, however, may not be the best indicator of disease as the soil samples were collected at point sources to represent a larger area over which the endemicity covers. The PPRMs showed that the HA of smectite, and kaolinite-smectite to a lesser degree, were higher than the HA of the minor phases, and of the positive controls. This is significant, as smectite was found to be one of the main contributors to podoconiosis prevalence in our recent geospatial study of soil characteristics in the same geographical area in Ethiopia ([9]). Smectites are known to be highly haemolytic in assays using sheep, bovine and human RBCs (e.g., [46],[47],[48],[49]). Other studies have shown the elevated HA of clays: kaolinite and bentonite (a montmorillonite-rich deposit, commonly formed from the weathering of volcanic ash) were found to be as haemolytic as crystalline silica (Min-U-Sil), whereas feldspar (a mineral commonly found in soil samples) was less haemolytic (e.g., [50]). Phyllosilicate minerals were also found to be cytotoxic in other in vitro assays, for example a recent study on rainbow trout gill epithelial cells established that the phyllosilicates, mica and kaolinite, were markedly more cytotoxic than the two framework silicates (quartz and feldspar) tested ([51]).

The elevated cytotoxic potential of phyllosilicate minerals is thought to be due to their high surface area, which is largely a consequence of their small particle size ([52]). In addition, the zeta potential of the individual mineral phases in the lymphatic fluid (at the appropriate pH) will determine the cytotoxic potential. Several studies have demonstrated that particle size is related to haemolytic activity. For example, the haemolytic potential of quartz particles increases with decreasing particle size ([53],[54]). Stronger cellular effects were noted for particles with larger surface areas when particles (of the same chemical composition) were introduced to human lung epithelial cells and alveolar macrophages ([55],[56]). Further to this, studies have noted that the haemolytic potential induced by larger agglomerates of silver nanoparticles is less than that induced by smaller agglomerates of the same composition ([57],[58]). In our study, the bivariate correlation between the proportion of particles <1 μm, after dispersion in water only and with deflocculant, and the haemolytic potential was weak (r = 0.089 and 0.156, respectively). However, further investigation of the correlation between haemolytic potential and zeta potential must be investigated, with additional measurement of the zeta potential of the individual mineral phases, and further consideration of analytical artifacts (such as dispersion inefficiencies, presence/absence of organic material/absorbance interferences etc.).

There is a complex control on HA in which no single variable (particle size, mineralogy of the particles, particle chemistry, particle surface charge) can explain the results. For further complexity, it has been noted that both false-positive and false-negative results in haemolysis may be caused by the presence of nanoparticles during haemolytic challenge (e.g., [59],[60]). Nanoparticles, such as gold-containing particles and some nano-emulsions, can result in false-positive readings if the nanoparticles have an absorbance close to or at the absorbance wavelength measured during haemolysis (i.e. ~550 nm) ([59]). When considering a single-phase, manufactured or synthesised nanoparticle sample, these properties may be identified, but in a multi-phase sample, such as a soil sample, these properties are more challenging to document. Iron oxides may interfere with absorbance at 550 nm, which may have contributed to our observation of a weak positive relationship between the quantity of iron oxide and the haemolytic potential of the soil. As the absorbance was measured over a range of soil concentrations, and with the absence of RBCs, interference is unlikely to be a major contributor in this case. However, complexation of various ions with the RBC surface, the leaching of metals from the soil sample surfaces and ion exchange effects were not measured in this study and may affect the outcome of the experiment.

In addition to the surface area (and size) of the particles, other factors have been shown to contribute to the cytotoxic potential of mineral particle ([52]). The morphology of the particles is important, and less rounded, acicular particles have been shown to elicit more cellular membrane damage than comparable rounded particles ([61]). The composition of the particle has an impact on the potential surface reactivity in vitro. In studies comparing pristine nanoclays with nanoclays that have been modified (exchange of the cations in the interlayer space, in this case modified with a ternary ammonium salt), the toxicity of the modified nanoclays was found to be greater than that of the pristine nanoclays. This held true in a selection of in vitro cellular systems ([62]).

We measured the ζP of the soils as a surrogate for the status of surface functional groups. The ζP of all the soil samples analyzed was negative, which is typical for clay-rich soils ([63]) and the majority of the PPRMs (except hematite) were also negative. The composition of the RBC membrane gives the surface a negative charge ([64]), and hence positively charged surfaces would be more likely to interact with RBCs. The lack of correlation between the ζP and the haemolytic response of the soil samples is also considered to be due to the multiple contributions from the mineral phases within the soils. However, in previous studies using single phase samples, the ζP (measured at pH 5.6) for a range of nanoparticles has been shown to correlate well with the haemolytic potential ([65]). This correlation, between ζP and haemolytic activity, has also been observed for asbestos minerals ([66]). Additionally, in experiments using polymeric nanoparticles with altered surface functionalities, the samples with a positive ζP were shown to have a haemolytic effect, whereas the samples with negative ζP showed no HA, explained by the fact that RBCs have a negative charge of -31.8 mV ([67]). When particles are incubated in biological fluids, a biomolecule corona (commonly proteins, lipids and carbohydrates; [68],[69],[70]) can cover the surface of the particle and alter its surface reactivity (for example by encouraging or moderating aggregation between particles, or suppressing the surface charge; [71]). Cho et al. ([67]) noted that the addition of a protein corona around polymeric nanoparticles altered the ζP from positive to negative and resulted in the loss of haemolytic potential. This is of interest to the study of podoconiosis as it is likely that particles within the soil enter the lymph system where they are able to react with biological structures. Light microscopy of lymphatic tissues showed that the birefringent particulate matter (predominantly silicates) was more common in areas of the tissue, and birefringence was more frequently observed in podoconiosis patients when compared with the control patients (without podoconiosis) ([72]). Further to this, Price and Henderson ([72]) hypothesised that the birefringence may indicate a protein coating around the particulate matter that may protect the surrounding tissues. However, the surface reactivity of mineral phases within the human body may alter over time, and Cho et al. ([65]) have demonstrated that the HA could be reinstated in metal oxide nanoparticles once digestive enzymes removed the protein corona.

Individual correlations between the HA and soil characteristics showed a number of associations, but no dominant feature was outstanding in the analysis. Multivariate analysis of the data is more appropriate in this instance, as it appears that the haemolysis assay is influenced by a number of factors. The predicted HA cannot be predicted reliably from the input parameters attained in this study. The PCA carried out here (Fig 6) identified two distinct groups of characteristics within the soil samples that, when entered into a regression model (Eq 3), helped to explain the variation in the normalised HA response that was elicited. The regression model notes that an increase in the normalized HA is caused by an increase in Y, Zr and Al_2_O_3_, and a concurrent increase in the amount of Fe_2_O_3_, TiO_2_, MnO and Ba in the soils. In our study, the quantity of TiO_2_, Al_2_O_3_, and MnO as separate phases in our soil samples was less than 5 wt. % as neither Ti, Al nor Mn oxide mineral phases were identified using X-ray diffraction. The link of Al to HA is speculated to be due to kaolinite. In addition, Y is likely concentrated by weathering processes–either due to cation exchange on the layer surfaces or chemisorption of anions at the layer edges—of phyllosilicates ([73]), for example, rare-earth hosting kaolinites termed “ionic clays” ([74]). Ti is likely present in some TiO_2_ phase in a low-concentration, as the association kaolinite-Ti oxides is typical ([75]). The Fe and Mn are likely concentrated in the iron oxide mineral structure (such as goethite), as could also happen with Ba. As previously suggested, the Zr (and Y) is likely to be found as zircons adhering to the phyllosilicate structure, as well as the phyllosilicates themselves. While associations exist between the elements and HA, it may be the presence of the mineral hosting the element that ultimately influences HA.

Although the mechanisms of nanoparticle toxicity are numerous and dependent on the type and chemistry of the particles, oxidative stress has been broadly implicated as an important driving mechanism behind inflammatory response and other adverse health impacts caused by nanoparticle exposure (e.g., [76]). In aqueous physiological media, such as body fluid, nanoparticles are thought to generate reactive oxygen species (ROS) which can have an impact on biochemical components such as proteins and polyunsaturated fatty acids due to the formation of free radicals ([77]). The influence of iron oxide on HA is potentially due to the redox-active nature of iron and iron-dependent free radical generation (via the Fenton reaction) that could contribute to the lysis of RBCs. In studies on the pathogenesis behind intravascular haemolysis, when FeCl_3_ was applied to a mouse aorta in the presence of perfused whole blood, the associated outcomes were endothelial denudation, collagen exposure and occlusive thrombus formation ([78]). Furthermore, Woollard et al. ([78]) hypothesize that within the vascular system the cycle of RBC lysis, hemoglobin release and subsequent oxidation can contribute to severe vascular denudation. TiO_2_ (both anatase and rutile) nanoparticles have been shown to impair cell function and specifically decrease cell area, proliferation, mobility and the functional ability to contract collagen (the latter is used to mimic the process of wound healing) and induce oxidative stress ([79]).

The majority of the in vitro toxicological assays carried out routinely analyze one single mineral or chemical phase as a method of predicting cellular damage. The multi-phase soil samples involved in this study are complex, and the outcome must be cautiously compared with single-phase in vitro assays due to the potential interaction between the phases that act to moderate the haemolytic activity. The composition of the particles that interact with biological structures, in particular the functional groups that are present on the surface of the mineral particles, will play an important role in determining the reactions that will occur ([80]). Michel et al. ([51]) established that the mechanism likely to be causing RBC lysis was markedly different for minerals of different compositions. Recent work investigating the haemolytic potential of various forms of silica (crystalline and amorphous) deduced that specific epitopes in the RBC membrane can strongly interact with dissociated or undissociated silanols and siloxane groups on the surface of the silica particles ([30]).

Further work will take into account the biopersistance and hardness of the minerals within the soil, which may contribute to the exacerbation of podoconiosis. Damage to the stratum corneum could be due to abrasion over time caused by repeated exposure to fine particles of quartz and other particles within the soil. The hygroscopicity of the soil would give an indication of its capacity to produce skin dehydration (leading to desiccation and cracking of the stratum corneum), and may be indicative of the swelling capacity of the clays once in the human body. Investigating the dissolution of the finer fractions of soil particles, and especially the nanoparticles, in different forms of physiological media will provide more information regarding the components within the soil that are likely to contribute to the initiation and/or progression of podoconiosis over time. Analysis of the soil samples, including the finer fractions, in cell lines are planned to explore the potential mechanistic pathways involved in podoconiosis pathogenesis.

## Conclusions

Haemolysis has previously been employed to predict the potential quartz-like inflammatory properties of environmental samples (such as respirable volcanic ash), and was employed in this study in an attempt to determine whether it would be a good indicator of toxicity in podoconiosis. As single phases, the minerals smectite and kaolinite-smectite (phyllosilicates identified and quantified in the soil samples) elicited a higher haemolytic responses than other single mineral phases tested. However, for the soil samples tested here, the haemolytic effect only slightly correlated with the presence of either smectite or smectite-kaolinite in the sample. This may suggest that there is a moderating effect in mixed phase (mineral) samples, where the haemolytic response is mediated in the presence of other mineral phases within the soil. The measured HA from the soil also did not correlate with the endemicity of podoconiosis as determined by where the samples were taken on the Ethiopian Volcanic Plateau. HA was found to increase with increasing proportions of Y, Zr, Al_2_O_3_, Fe_2_O_3_, TiO_2_, MnO and Ba in the soils. The driving factors for HA are complex in multiphase samples. It is likely that this study only represents a snapshot of in vitro reactivity in time, and further work must address the biopersistance of these mineral phases within the skin substructure. In addition, future work must address the characteristics of the nanoscale particles within the soils, as it is likely that only the finest fraction of the soil is able to penetrate the stratum corneum and elicit damage.
